# Anti-inflammatory properties of a dual PPARgamma/alpha agonist muraglitazar in *in vitro *and *in vivo *models

**DOI:** 10.1186/ar4211

**Published:** 2013-04-17

**Authors:** Erja-Leena Paukkeri, Tiina Leppänen, Mira Lindholm, Mun Fei Yam, Mohd Zaini Asmawi, Anne Kolmonen, Paula H Aulaskari, Eeva Moilanen

**Affiliations:** 1 The Immunopharmacology Research Group, University of Tampere School of Medicine and Tampere University Hospital, Medisiinarinkatu 3, Tampere, FI-33014, Finland; 2 School of Pharmaceutical Sciences, Universiti Sains Malaysia, Minden, MY-11800, Pulau Pinang, Malaysia; 3 Department of Chemistry, University of Eastern Finland, Joensuu Campus, Yliopistokatu 7, Joensuu, FI-80101, Finland

## Abstract

**Introduction:**

Peroxisome proliferator-activated receptor (PPAR) agonists are widely used drugs in the treatment of diabetes and dyslipidemia. In addition to their metabolic effects, PPAR isoforms PPARα and PPARγ are also involved in the regulation of immune responses and inflammation. In the present study, we investigated the effects of a dual PPARγ/α agonist muraglitazar on inflammatory gene expression in activated macrophages and on carrageenan-induced inflammation in the mouse.

**Methods:**

J774 murine macrophages were activated by lipopolysaccharide (LPS) and treated with dual PPARγ/α agonist muraglitazar, PPARγ agonist GW1929 or PPARα agonist fenofibrate. The effects of PPAR agonists on cytokine production and the activation of inducible nitric oxide synthase (iNOS) pathway were investigated by ELISA, Griess method, Western blotting and quantitative RT-PCR. Nuclear translocation, DNA-binding activity and reporter gene assays were used to assess the activity of nuclear factor kappa B (NF-kB) transcription factor. Carrageenan-induced paw oedema was used as an *in vivo *model of acute inflammation.

**Results:**

Muraglitazar as well as PPARγ agonist GW1929 and PPARα agonist fenofibrate inhibited LPS-induced iNOS expression and NO production in activated macrophages in a dose-dependent manner. Inhibition of iNOS expression by muraglitazar included both transcriptional and post-transcriptional components; the former being shared by GW1929 and the latter by fenofibrate. All tested PPAR agonists also inhibited IL-6 production, while TNFα production was reduced by muraglitazar and GW1929, but not by fenofibrate. Interestingly, the anti-inflammatory properties of muraglitazar were also translated *in vivo*. This was evidenced by the finding that muraglitazar inhibited carrageenan-induced paw inflammation in a dose-dependent manner in mice as did iNOS inhibitor L-NIL and anti-inflammatory steroid dexamethasone.

**Conclusions:**

These results show that muraglitazar has anti-inflammatory properties both *in vitro *and *in vivo *and these effects reflect the agonistic action through both PPARα and PPARγ.

## Introduction

It has long been known that macrophages are pivotal cells in the pathogenesis of autoimmune diseases, including rheumatoid arthritis [1,2]. During the last decades macrophages have also been found to affect metabolism in various tissues and today it is widely agreed that macrophages are also associated with the pathophysiology of many obesity-linked diseases [3-5]. The mechanisms connecting macrophage activation and metabolism are not fully known but some hypotheses have been proposed. Macrophages are present in adipose tissue and when aberrantly activated in relation to obesity they produce inflammatory factors by themselves and also activate surrounding adipocytes to release signalling proteins called adipokines. Adipokines are known to regulate energy metabolism and appetite but also inflammatory responses, arthritis and catabolic processes in articular cartilage [6]. It is also obvious that obesity-related changes in energy metabolism directly regulate macrophage responses [7]. From the pharmacological point of view, an interesting question exists of if or how the macrophage phenotype and secretory profile differ between chronic inflammation typical, for example, in rheumatoid arthritis and obesity-related systemic inflammation, and if the same known and future anti-inflammatory compounds would have therapeutic value in only one or both of those inflammatory conditions.

Peroxisome proliferator-activated receptors (PPARs) are members of the nuclear receptor superfamily and they are strongly linked to the regulation of energy homeostasis in cells. PPARs are expressed in three isoforms: PPARα, PPARγ and PPARβ/δ. PPARγ agonists thiazolidinediones (TZDs) and PPARα agonist fibrates are widely used as pharmacological agents in the treatment of diabetes and dyslipidemia, respectively. Although the main interest in PPAR-related studies has been focused on the role of PPARs in energy homeostasis, PPARs, especially PPARα and PPARγ, are also shown to be involved in the regulation of the immune and inflammatory responses in obesity-linked diseases [8-10]. During the last few years some reports have been published showing that PPARγ agonists reduce inflammatory responses in animal models of rheumatoid arthritis [11]. Nevertheless, we found only a single study suggesting that also a PPARα agonist fenofibrate would have anti-inflammatory effects in experimentally induced arthritis [12].

Muraglitazar is a dual PPARγ/α-agonist that has strong PPARγ and moderate PPARα effects [13]. Originally, it was developed for the treatment of type II diabetes with a view to combining both insulin sensitizing and antihyperlipidemic effects of the PPAR agonists. Muraglitazar has been shown to decrease the levels of HbA_1c_, FFA and triglycerides and to increase the levels of HDL in humans when compared to placebo, and it appeared to be more potent than pioglitazone [14,15]. However, despite the fact that there are several reports on the antidiabetic effects of muraglitazar, there are no previous studies on the effects of muraglitazar in inflammatory processes.

In the present study, we aimed to investigate the anti-inflammatory effects of muraglitazar. Since the immunoregulatory effects of PPARα and PPARγ are somewhat different, we wanted to study whether muraglitazar has more potent anti-inflammatory effects than PPARγ or PPARα agonists alone. To examine this we investigated the effects of muraglitazar on the activation of the inducible nitric oxide synthase (iNOS) pathway and on the production of inflammatory cytokines in activated macrophages. We extended our study by testing the effect of muraglitazar on carrageenan-induced paw inflammation in the mouse.

## Materials and methods

### Materials

Reagents were obtained as follows: GW1929 and MG132 from Tocris Bioscience (Bristol, UK), N^6^-(1-iminoethyl)-L-lysine (L-NIL) from Enzo Life Sciences Ltd. (Exeter, UK), rabbit polyclonal β-actin, lamin A/C and iNOS antibodies and goat HRP-conjugated anti-rabbit polyclonal antibody from Santa Cruz Biotechnology Inc. (Santa Cruz, CA, USA). Nuclear factor kappa B (NF-κB) subunit p65 antibody was from Cell Signaling Technology Inc. (Danvers, MA, USA). Muraglitazar was synthesised in the laboratory of Dr. Paula H. Aulaskari (University of Eastern Finland, Joensuu Campus, Joensuu, Finland), see below. Other reagents were from Sigma-Aldrich Co. (St. Louis, MO, USA).

Muraglitazar was synthesised by a five-step procedure according to Devasthale *et al*. [13]. The structure and purity of intermediates and the final product muraglitazar were confirmed by melting point analysis (Gallenkamp melting point apparatus MFB-595, Gallenkamp, Loughborough, UK), ^1^H, ^13^C NMR spectroscopy (Bruker Avance 250 MHz and 400 MHz spectrometer, Bruker BioSpin AG, Fällanden, Switzerland) using deuteriumchloroform as a solvent and tetramethylsilane as a reference, and by IR spectroscopy (Nicolet Avatar 320 FT-IR spectrometer, Thermo Electron Scientific Instruments, LLC, Madison, WI, USA) using dry potassium bromide as a salt component of solid mixture. The molecular structure and the purity of muraglitazar were also confirmed by mass spectrometer and elemental analysis. The mass spectrometer measurements were performed on Bruker BioAPEX II 47e Fourier transform ion cyclotron resonance (FTICR) mass spectrometer (Bruker Daltonics, Billerica, MA, USA) equipped with an InfinityTM cell, 4.7 Tesla 160-mm-bore superconducting magnet (Magnex Scientific Ltd., Abingdon, UK), and an external electron ionization (EI) or electrospray ion source (ESI) (Analytica of Branford Inc., Branford, CT, USA). Elemental analysis was performed on CE Instruments EA 1110 elemental analyser (CE Instruments Ltd., Rodano, MI, Italy). The results of elemental analysis were within ± 0.2% of the theoretical values.

### Cell culture

Murine J774 macrophages (American Type Culture Collection, Manassas, VA, USA) were cultured at 37°C in 5% CO_2 _atmosphere in Dulbecco's modified Eagle's medium with Ultraglutamine 1 (Lonza Group Ltd., Basel, Switzerland) supplemented with 10% heat-inactivated foetal bovine serum (Lonza Group Ltd), 100 U/ml penicillin, 100 µg/ml streptomycin and 250 ng/ml amphotericin B (Invitrogen Co., Carlsbad, CA, USA) and harvested with trypsin-EDTA (Invitrogen Co.). Cells were seeded on 24-well plates for RNA extraction and nitrite and ELISA measurements, on 24-well plates or 6-well plates for preparation of cell lysates for Western blotting, on 10 cm dishes for preparation of nuclear extracts for Western blotting and NF-κB p65 DNA binding assay and on 96-well plates for an XTT test. Confluent cultures were exposed to fresh culture medium containing the compounds of interest.

Human HEK293 cells (American Type Culture Collection) were cultured at 37°C in 5% CO_2 _atmosphere in Eagle's minimal essential medium (Lonza Group Ltd.) supplemented with 0.15% sodium bicarbonate, 100 µM non-essential aminoacids, 1 mM sodium pyruvate, 10% heat-inactivated foetal bovine serum (Lonza Group Ltd), 100 U/ml penicillin, 100 µg/ml streptomycin and 250 ng/ml amphotericin B (Invitrogen Co.) and harvested with trypsin-EDTA (Invitrogen Co.). Cells were seeded on 24-well plates for RNA extraction. Confluent cultures were exposed to fresh culture medium containing the compounds of interest.

Cell viability after treatment with combinations of LPS or cytokine mixture and the tested compounds was assessed by modified XTT test (Cell Proliferation Kit II, Roche Diagnostics, Mannheim, Germany) according to the manufacturer's instructions.

### Preparation of stable J774-pGL4(miNOS-prom)neo and HEK293-pNF-κB(luc)neo reporter cell lines

The pGL-MNOS II-5'-Luc plasmid [16] containing 5'-flanking sequence (1,171 bp, positions -1,570 to +141; promoter and a part of exon 1) of the murine iNOS gene was provided by Professor Harmut Kleinert (Johannes Gutenberg University, Mainz, Germany). This plasmid was digested with *Kpn*I and *Hind*III, and the restriction fragment, containing murine iNOS promoter and part of exon 1, was then cloned into the *Kpn*I/*Hind*III site of firefly luciferase reporter plasmid pGL4.17(luc2/neo) (Promega, Madison, WI, USA) generating pGL4(miNOS-prom)neo, in which the luciferase gene is driven by murine iNOS promoter. The plasmid was sequenced to confirm the appropriate size, position and orientation of the insert in the plasmid. To create a stable transfection with murine iNOS promoter reporter plasmid, J774 cells were transfected with pGL4(miNOS-prom)neo using Lipofectamine 2000 (Invitrogen Co.) according to the manufacturer's instructions. Transfected cells were selected with G418 disulfate salt (Sigma-Aldrich Co.) treatment (800 µg/ml). After the selection, the survived clones were pooled to give rise to J774-pGL4(miNOS-prom)neo cell line and further cultured in the presence of 400 µg/ml of G418.

To create a stable transfection with NF-κB reporter plasmid, HEK293 cells were transfected with pGL4.32[luc2P/NF-κB-RE/Hygro] (Promega) using Lipofectamine 2000 (Invitrogen Co.) according to the manufacturer's instructions. Transfected cells were selected with hygromycin B (EMD Biosciences Inc., La Jolla, CA, USA) treatment (200 µg/ml). After the selection, the survived clones were pooled to give rise to HEK293- pGL4.32[luc2P/NF-κB-RE/Hygro] cell line and further cultured in the presence of 100 µg/ml of hygromycin B.

### Nitrite determination

Nitrous oxide (NO) production was determined by measuring the accumulation of nitrite, a stable metabolite of NO in aqueous condition, into the culture medium. The culture medium was collected at indicated time points and nitrite was measured by the Griess reaction [17].

### Preparation of cell lysates for Western blotting

At indicated time points, the cells were rapidly washed with ice-cold phosphate-buffered saline and solubilized in cold lysis buffer containing 10 mM Tris-base, pH 7.4, 5 mM EDTA, 50 mM NaCl, 1% Triton X-100, 0.5 mM phenylmethylsulphonyl fluoride, 1 mM sodium orthovanadate, 20 μg/ml leupeptin, 50 μg/ml aprotinin, 5 mM NaF, 2 mM sodium pyrophosphate and 10 μM n-octyl-β-D-glucopyranoside. After incubation on ice for 15 minutes, lysates were centrifuged (13,400 *g*, 4°C, 10 minutes), supernatants were collected and mixed 3:1 with SDS sample buffer (62.5 mM Tris-HCl, pH 6.8, 10% glycerol, 2% SDS, 0.025% bromophenol blue and 5% β-mercaptoethanol). The samples were stored at -20°C until analysed. An aliquot of the supernatant was used to determine protein concentration by the Coomassie blue method [18].

### Preparation of nuclear extracts for Western blotting

At indicated time points, the cells were rapidly washed with ice-cold phosphate-buffered saline and solubilized in hypotonic buffer A (10 mM HEPES-KOH, pH 7.9, 1.5 mM MgCl_2_, 10 mM KCl, 0.5 mM dithiothreitol, 0.2 mM phenylmethylsulphonyl fluoride, 1 mM sodium orthovanadate, 10 μg/ml leupeptin, 25 μg/ml aprotinin, 1 mM NaF and 0.1 mM EGTA). After incubation on ice for 10 minutes, the cells were vortexed for 30 seconds and the nuclei were separated by centrifugation at 4°C, 21,000*g *for 10 seconds. Nuclei were resuspended in buffer C (20 mM HEPES-KOH, pH 7.9, 25% glycerol, 420 mM NaCl, 1.5 mM MgCl_2_, 0.5 mM dithiothreitol, 0.2 mM phenylmethylsulphonyl fluoride, 1 mM sodium orthovanadate, 10 μg/ml leupeptin, 25 μg/ml aprotinin, 1 mM NaF and 0.1 mM EGTA) and incubated on ice for 20 minutes. Nuclei were vortexed for 30 seconds and nuclear extracts were obtained by centrifugation at 4°C, 21,000 *g *for 2 minutes. Supernatants were collected and mixed 3:1 with SDS sample buffer. The samples were stored at -70°C until analysed. Coomassie blue method was used to measure the protein content of the samples [18].

### Western blotting

Prior to Western blotting, samples were boiled for 10 minutes and 20 μg of protein was loaded per lane on 10% or 12% SDS-polyacrylamide gels and separated by electrophoresis. Proteins were transferred to Hybond enhanced chemiluminescence nitrocellulose membrane (GE Healthcare, Little Chalfont, Buckinghamshire, UK). After the transfer, the membrane was blocked in TBS/T (20 mM Tris-base pH 7.6, 150 mM NaCl, 0.1% Tween-20) containing 5% of non-fat dry milk or 5% bovine serum albumin at room temperature for one hour and incubated with primary antibody in the blocking solution at 4°C overnight. The membrane was washed with TBS/T, incubated with secondary antibody in the blocking solution at room temperature for one hour and washed. Bound antibody was detected using SuperSignal West Pico or Dura chemiluminescent substrate (Pierce, Rockford, IL, USA) and ImageQuant LAS 4000 mini imaging system (GE Healthcare). The chemiluminescent signal was quantified with ImageQuant TL 7.0 image analysis software.

### RNA extraction and quantitative real-time PCR

At the indicated time points, culture medium was removed and total RNA of the cultured cells was extracted using GenElute™ Mammalian Total RNA Miniprep kit (Sigma-Aldrich Co.). For luciferase mRNA experiments, total RNA was treated with DNase I (Fermentas UAB, Vilnius, Lithuania). Total RNA (100 ng) was reverse-transcribed to cDNA using TaqMan Reverse Transcription reagents and random hexamers (Applied Biosystems, Foster City, CA, USA). cDNA obtained from the reverse transcription reaction was diluted 1:20 with RNase-free water and was subjected to quantitative PCR using TaqMan Universal PCR Master Mix and ABI Prism 7000 sequence detection system (Applied Biosystems).

Total RNA of the tissue samples was extracted by GenElute™ Mammalian Total RNA Miniprep kit with proteinase K digestion (Sigma-Aldrich Co.). Total RNA (500 ng) was reverse-transcribed to cDNA using Maxima First Strand cDNA Synthesis Kit (Fermentas UAB). cDNA obtained from the reverse transcription reaction was diluted 1:20 with RNase-free water and was subjected to quantitative PCR using TaqMan Universal PCR Master Mix and ABI Prism 7000 sequence detection system (Applied Biosystems).

Primers and probes for luciferase, IL-6, iNOS and glyceraldehyde-3-phosphate dehydrogenase (GAPDH) (Table 1) were optimized according to the manufacturer's instructions in TaqMan Universal PCR Master Mix Protocol part number 4304449 revision C. The expression of mouse TNFα mRNA was measured by using TaqMan^® ^Gene Expression Assay (Mm00443260_g1, Applied Biosystems, Foster City, CA, USA).

**Table 1:** Primer and probe sequences.

**Gene**	**Oligonucleotide**	**Sequence 5' → 3'**
Human GAPDH	Forward primer	TCCTACCACCAGCAACCCTGCCA
	Reverse primer	GCAACAATATCCACTTTACCAGAGTTAA
	Probe	CGCCTGGTCACCAGGGCTGC
Luciferase	Forward primer	ACGGCTTCGGCATGTTCA
	Reverse primer	CTCCTCCTCGAAGCGGTACA
	Probe	TTGATCTGCGGCTTTCGGGTCGT
Mouse GAPDH	Forward primer	GCATGGCCTTCCGTGTTC
	Reverse primer	GATGTCATCATACTTGGCAGGTTT
Mouse IL-6	Probe	TCGTGGATCTGACGTGCCGCC
	Forward primer	TCGGAGGCTTAATTACACATGTTC
	Reverse primer	CAAGTGCATCATCGTTGTTCATAC
	Probe	CAGAATTGCCATTGCACAACTCTTTTCTCA
Mouse iNOS	Forward primer	CCTGGTACGGGCATTGCT
	Reverse primer	GCTCATGCGGCCTCCTT
	Probe	CAGCAGCGGCTCCATGACTCCC

PCR reaction parameters were as follows: incubation at 50°C for 2 minutes, incubation at 95°C for 10 minutes, and thereafter 40 cycles of denaturation at 95°C for 15 s and annealing and extension at 60°C for 1 minute. Each sample was determined in duplicate.

The relative mRNA levels were quantified and compared using the relative standard curve method as described in Applied Biosystems User Bulletin number 2.

### NF-κB p65 DNA binding assay

DNA binding activity of NF-κB p65 was evaluated using NF-κB (p65) Transcription Factor Assay Kit (Cayman Chemical Company, Ann Arbor, MI, USA). The nuclear extracts for the assay were prepared according to the manufacturer's instructions and 10 µg of nuclear protein per well was used for the experiment. The samples were incubated at +4°C overnight with the dsDNA templates carrying NF-κB response element. After primary (anti-NF-κB p65) and secondary (goat anti-rabbit HRP) antibody treatments, developing reagents were added and absorbance was read at 450 nm.

### Enzyme-Linked Immunosorbent Assay (ELISA)

Culture medium samples were kept at -20°C until assayed. The concentrations of IL-6 and TNFα in culture medium were determined by ELISA according to the manufacturer's instructions (R&D Systems Europe, Abingdon, UK).

### Carrageenan-induced paw oedema in the mouse

Prior to the experiment investigating the time dependent formation of carrageenan-induced paw oedema, 36 male Charles River mice (weighing 25.0 to 30.0 g) were housed and cared for under the guidelines of the institutional animal care and use committee with food and water provided *ad libitum*. The study was approved by the Animal Ethic Committee, Universiti Sains Malaysia.

The animals were randomly divided into six groups (six mice per group) and each group was treated orally with the test compound suspended in 2.0% (w/v) carboxymethyl cellulose (CMC) in distilled water. The test groups were as follows: control (treated with 2.0% CMC only), muraglitazar 12.5 mg/kg, muraglitazar 25 mg/kg, muraglitazar 50 mg/kg, L-NIL 50 mg/kg and dexamethasone 2 mg/kg. An hour after the treatment, 40 µl of carrageenan (1% suspension in normal saline) was injected subcutaneously into the left hind paws of the animals. Thicknesses of the paws were measured by micrometer one hour before and one hour, four hours and six hours after the carrageenan injection. The results are expressed as percentages of swelling calculated as follows:

$$\begin{array}{l} \text{Difference}=\text{(thickness of the hind paw at indicated time point}-\text{thickness of the hind }\\ \text{paw before carrageenan)}/\text{thickness of the hind paw before carrageenan.} \end{array}$$

The carrageenan-induced gene expression was investigated in C57BL/6 mice. The study was approved by the Animal Care and Use Committee of the University of Tampere and the respective provincial committee for animal experiments. Animals were housed under standard conditions of light, temperature and humidity (12:12 h light-dark cycle, 22 ± 1°C, 50 to 60%) with food and water provided *ad libitum*.

Mice were randomly divided into two study groups with five and seven mice in the groups and muraglitazar 50 mg/kg was injected intraperitoneally into the group of five mice. Two hours after the treatment, the mice were anesthetized with an intraperitoneal injection of 0.5 mg/kg of medetomidine (Orion Oyj, Espoo, Finland) and 75 mg/kg of ketamine (Pfizer Oy Animal Health, Helsinki, Finland), and 30 µl of λ-carrageenan (1.5% suspension in normal saline) was injected subcutaneously into a hind paw of the animals. As a control, 30 µl of saline was injected into the contralateral paws. The paw volumes were measured before and six hours after carrageenan injection by plethysmometer (Ugo Basile Srl, Comerio, Italy). Oedema is expressed as the difference between the change in carrageenan-treated paw volume and the control paw volume in µl. After six hours of carrageenan injection, the mice were sacrificed by cervical dislocation. Carrageenan-treated and control paws were skinned and the soft tissues of the paws were collected and RNA was extracted as described above.

### Statistics

Results are expressed as mean + standard error of mean (SEM). When indicated, statistical significance was calculated by analysis of variance followed by Dunnett's multiple comparisons test or Mann-Whitney test. Differences were considered significant at *P *<0.05.

## Results

### Muraglitazar *and PPARα and PPARγ agonists decreased NO production and iNOS expression*

**Figure 1:**
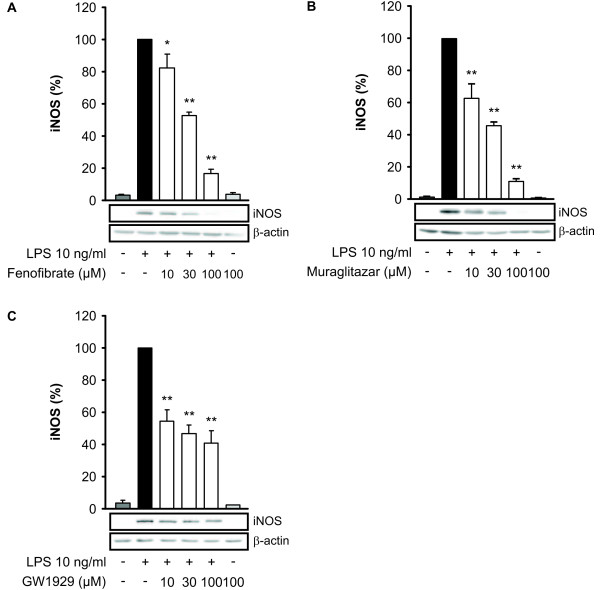
**Effects of PPAR agonists on iNOS protein expression in J774 macrophages**. Cells were stimulated with lipopolysaccharide (LPS) and treated with increasing concentrations of fenofibrate (**a**), muraglitazar (**b**) or GW1929 (**c**). After 24 h incubation, proteins were extracted and the levels of inducible nitric oxide synthase (iNOS) protein were analysed by Western blotting. β-actin was used as a loading control. Results represent the mean + SEM of three experiments, from which one representative Western blot is shown. *P <0.05 and **P <0.01 as compared to cells treated with LPS alone.

**Figure 2:**
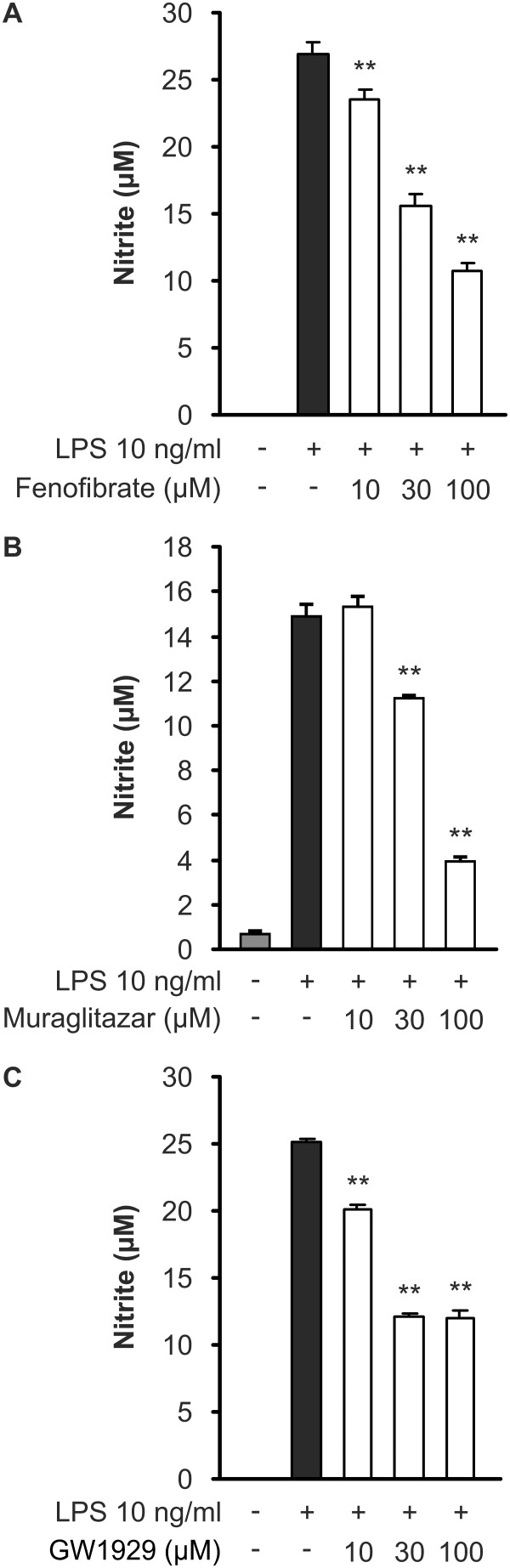
**Effects of PPAR agonists on NO production in J774 macrophages**. Cells were stimulated with lipopolysaccharide (LPS) and treated with increasing concentrations of fenofibrate (**a**), muraglitazar (**b**) or GW1929 (**c**). After 24 h incubation, nitrite accumulated into the culture medium was measured by Griess reaction as a marker of nitric oxide (NO) production. Results represent the mean + SEM (*n *= 6). **P <0.01 as compared to cells treated with LPS alone.

Resting J774 macrophages did not produce detectable amounts of NO, but when the cells were activated through TLR4 pathway by bacterial endotoxin LPS, NO production and iNOS expression were increased. Muraglitazar, PPARα agonist fenofibrate and PPARγ agonist GW1929 decreased LPS-induced iNOS expression (Figure 1) and NO production (Figure 2) in a dose-dependent manner. Muraglitazar, fenofibrate or GW1929 did not affect cell viability at the concentration used as determined by XTT test.

### Muraglitazar and a PPARγ agonist, but not a PPARα agonist, reduced iNOS mRNA expression

**Figure 3:**
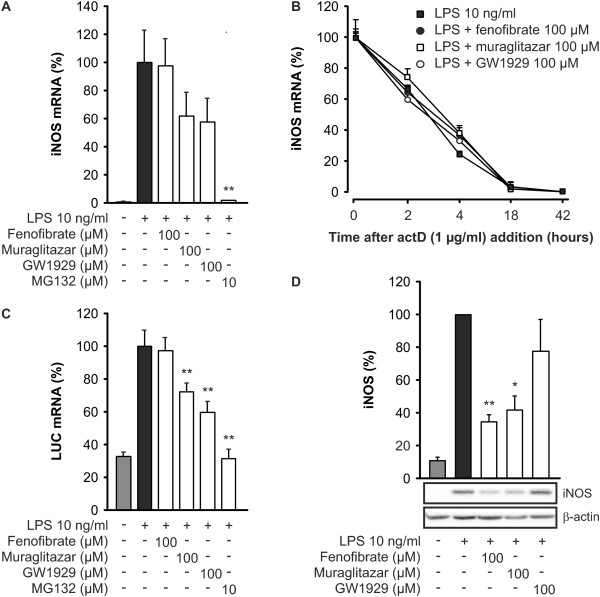
**Effects of PPAR agonists on iNOS expression in activated J774 macrophages**. (**a**) Effects of peroxisome proliferator-activated receptor (PPAR) agonists on inducible nitric oxide synthase (iNOS) mRNA expression. Cells were treated with PPAR agonists, NF-κB inhibitor MG132 or vehicle (dimethyl sulfoxide, DMSO) and stimulated with LPS for six hours. Total RNA was extracted and iNOS mRNA was determined by RT-PCR. The results were normalized against GAPDH mRNA. (**b**) Effects of PPAR agonists on iNOS mRNA degradation. Cells were treated with PPAR agonists or vehicle (DMSO) and stimulated with lipopolysaccharide (LPS). After six hours incubation, actinomycin D (1 µg/ml) was added to the cell culture to stop transcription. Total RNA was extracted at indicated time points after actinomycin D addition, and iNOS mRNA was determined by RT-PCR. The results were normalized against GAPDH mRNA. (**c**) Effects of PPAR agonists on iNOS promoter activity in J774 macrophages stably transfected with a luciferase (LUC) reporter gene under the control of iNOS protein. Cells were treated with PPAR agonists, NF-κB inhibitor MG132 or vehicle (DMSO) and stimulated with LPS for six hours. Total RNA was extracted and LUC mRNA was determined by RT-PCR. The results were normalized against GAPDH mRNA. (**d**) Post-transcriptional effects of PPAR agonists on iNOS protein levels. Cells were stimulated with LPS for 10 hours. After the stimulation, the culture medium was changed and the cells were further incubated with PPAR agonists or vehicle (DMSO) without LPS for additional 14 hours. Then, proteins were extracted and the levels of iNOS protein were analysed by Western blotting. β-actin was used as a loading control. Results represent the mean + SEM (*n *= 3 to 4). In d one representative Western blot is shown. *P <0.05 and **P <0.01 as compared to cells treated with LPS alone.

Since all three PPAR agonists decreased iNOS expression and NO production, we went further and determined the effects of PPAR agonists on iNOS mRNA expression. Muraglitazar and GW1929 reduced the levels of iNOS mRNA when determined six hours after the addition of LPS. However, fenofibrate had no effect on iNOS mRNA levels as compared to the cells treated with LPS only. NF-κB inhibitor MG132 was used as a control compound and it reduced LPS-induced iNOS mRNA levels as expected (Figure 3a).

Since the reduction of iNOS mRNA levels may be a sign of either transcriptional inhibition or increase in iNOS mRNA degradation, we studied the effect of PPAR agonists on the half-life of iNOS mRNA. After six-hour incubation with LPS alone or together with PPAR agonists, actinomycin D was added to the cells to stop mRNA synthesis. Incubations were terminated at different time points after the addition of actinomycin D. None of the PPAR agonists did affect the stability of iNOS mRNA when compared to the cells treated with LPS only (Figure 3b). These results suggest that the suppressive effect of muraglitazar and GW1929 on LPS-induced iNOS mRNA levels is mediated at the level of iNOS transcription.

We further investigated the effect of muraglitazar on the activity of iNOS promoter in J774 macrophages stably transfected to express luciferase reporter gene under the control of full length murine iNOS promoter. Similar to the effects on iNOS mRNA levels in wild-type J774 cells, muraglitazar and GW1929, but not fenofibrate, reduced LPS-induced iNOS promoter activity (Figure 3c).

The different effects of fenofibrate and GW1929 on iNOS mRNA expression may be explained by our previous findings that PPARα agonists regulate iNOS expression at the post-transcriptional level [19]. To study the post-transcriptional effects of muraglitazar and the other two PPAR agonists on iNOS protein levels, we first stimulated J774 macrophages with LPS for 10 hours, and PPAR agonists were added thereafter for 14 hours. Muraglitazar and fenofibrate, but not GW1929, were able to reduce iNOS protein levels in these post-transcriptional time points (Figure 3d).

### Effects of muraglitazar and PPARα and PPARγ agonists on TNFα and IL-6 production

**Figure 4:**
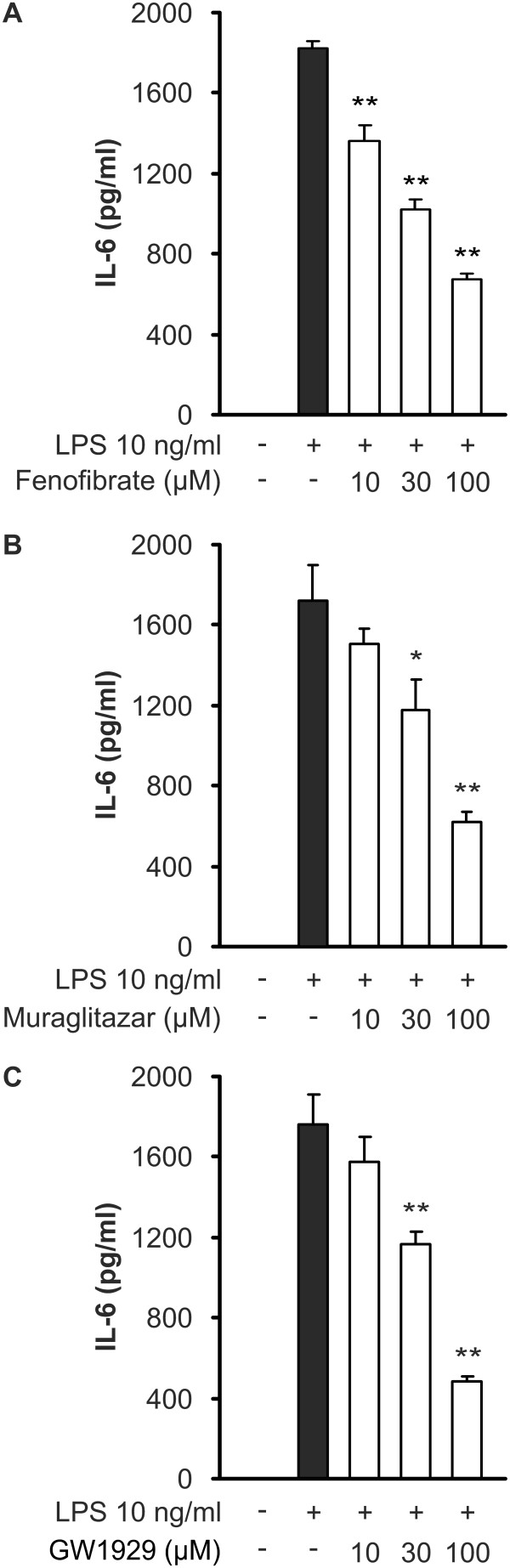
**Effects of PPAR agonists on IL-6 production in J774 macrophages**. Cells were treated with increasing concentrations of fenofibrate (**a**), muraglitazar (**b**) or GW1929 (**c**) and stimulated with lipopolysaccharide (LPS). After 24 h incubation, IL-6 accumulated into the culture medium was measured by ELISA. Results represent the mean + SEM (*n *= 4). *P <0.05 and **P <0.01 as compared to cells treated with LPS alone.

**Figure 5:**
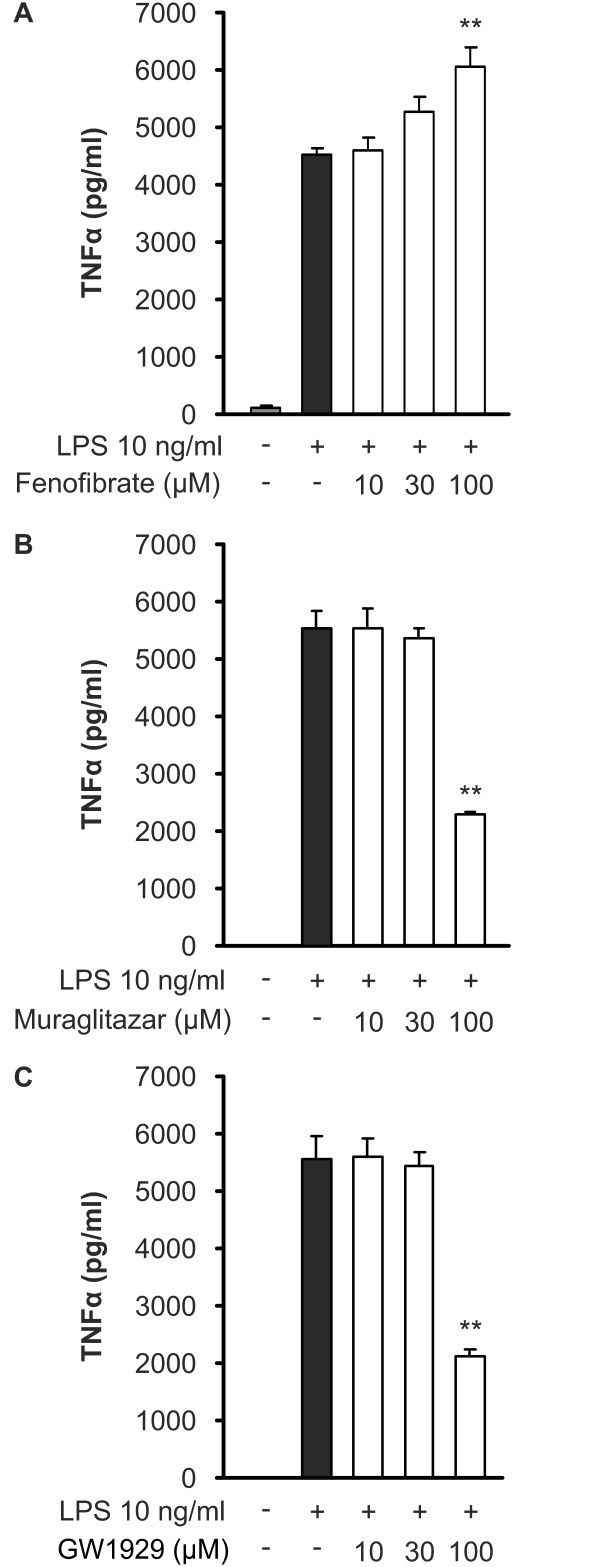
**Effects of PPAR agonists on TNFα production in J774 macrophages**. Cells were treated with increasing concentrations of fenofibrate (**a**), muraglitazar (**b**) or GW1929 (**c**) and stimulated with lipopolysaccharide (LPS). After 24 h incubation, TNFα accumulated into the culture medium was measured by ELISA. Results represent the mean + SEM (*n *= 4). *P <0.05 and **P <0.01 as compared to cells treated with LPS alone.

In order to investigate if PPAR agonists regulate the expression of other genes related to innate immunity, we measured their effects on IL-6 and TNFα production. LPS induced TNFα and IL-6 production in J774 cells when measured with ELISA in the culture medium after 24 hours incubation. All the agonists reduced IL-6 production in a dose-dependent manner (Figure 4). However, LPS-induced TNFα production was reduced only by GW1929 and muraglitazar at the highest concentration used (Figure 5c, b). Fenofibrate, on the other hand, tended to increase TNFα production at higher concentrations (Figure 5a).

### Muraglitazar or PPARα or PPARγ agonists had no effect on NF-κB activation

As we have reported in this paper, muraglitazar reduces the synthesis of iNOS, IL-6 and TNFα. The syntheses of all these inflammatory markers are regulated by transcription factor NF-κB [20,21]. Therefore, we hypothesized that muraglitazar might affect the activity of NF-κB.

**Figure 6:**
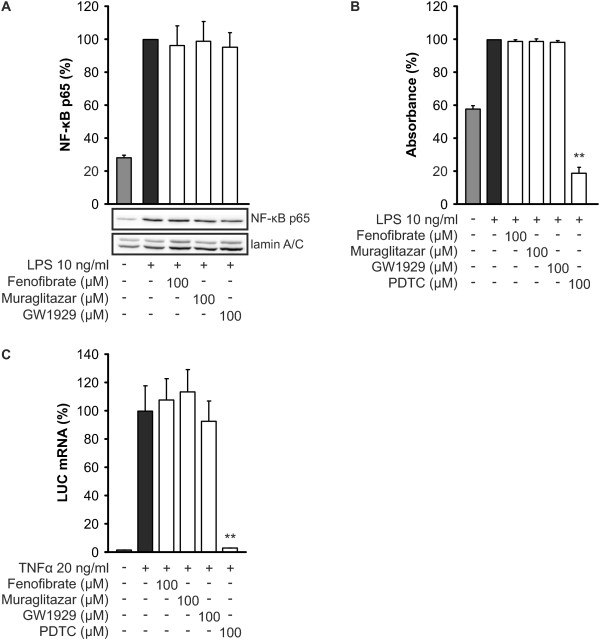
**Effects of PPAR agonists on NF-κB activation and NF-κB-mediated transcription**. (**a**) Effects of peroxisome proliferator-activated receptor (PPAR) agonists on NF-κB translocation to nucleus. J774 cells were preincubated with PPAR agonists or vehicle (dimethyl sulfoxide, DMSO) for two hours before addition of lipopolysaccharide (LPS). After 30 minutes incubation with LPS, nuclear extracts were prepared. The levels of NF-κB p65 in nuclear extracts were analysed by Western blotting. Lamin A/C was used as a loading control. (**b**) Effects of PPAR agonists on DNA binding activity of NF-κB. J774 cells were preincubated with PPAR agonists, NF-κB inhibitor pyrrolidine dithiocarbamate (PDTC) or vehicle (DMSO) for two hours before addition of LPS. After one hour incubation with LPS, nuclear extracts were prepared and the binding activity of NF-κB p65 to DNA was analysed as described in methods. (**c**) Effects of PPAR agonists on NF-κB-mediated transcription activity. HEK293 cells were stably transfected with a luciferase (LUC) reporter gene under the control of NF-κB-responsive promoter. The cells were preincubated with PPAR agonists, NF-κB inhibitor PDTC or vehicle (DMSO) for two hours before addition of TNFα. After six hours' incubation with TNFα, total RNA was extracted and LUC mRNA was determined by RT-PCR. The LUC mRNA levels were normalized against GAPDH mRNA. Results represent the mean + SEM (*n *= 3 to 4). In a one representative Western blot is shown. **P <0.01 as compared to cells treated with LPS or TNFα alone.

To test this, we first investigated the effect of muraglitazar on the nuclear translocation of NF-κB. LPS enhanced the nuclear translocation of NF-κB peaking at 30 minutes of stimulation. Muraglitazar, fenofibrate or GW1929 did not affect the nuclear levels of NF-κB p65 when compared to cells treated with LPS only (Figure 6a).

We continued by studying whether PPAR agonists affect the DNA binding activity of NF-κB. As illustrated in Figure 6b, muraglitazar, fenofibrate or GW1929 did not reduce the ability of NF-κB to bind to dsDNA fragments containing NF-κB response element. NF-κB inhibitor pyrrolidine dithiocarbamate (PDTC) was used as a positive control compound and it reduced LPS-induced NF-κB binding as expected.

Further, we evaluated the effect of PPAR agonists on NF-κB -mediated transcription using HEK293 cells stably transfected with a luciferase reporter gene under the control of NF-κB-responsive promoter. PPAR agonists did not modulate NF-κB-mediated transcription while NF-κB inhibitor PDTC had an effect as expected (Figure 6c).

### Muraglitazar reduced carrageenan-induced inflammatory responses in the mouse

**Figure 7:**
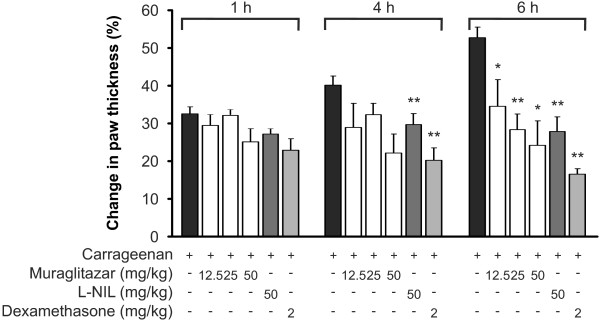
**Effect of muraglitazar on carrageenan-induced paw oedema in the mouse**. Muraglitazar, inducible nitric oxide synthase (iNOS) inhibitor L-NIL, dexamethasone or vehicle (2.0% CMC) were given to animals per os 1 hour prior to carrageenan injection (1%). Paw oedema was measured before and one, four and six hours after carrageenan injection by micrometer. Oedema is expressed as increase in paw thickness at indicated time points compared to thickness before carrageenan. Values are mean + SEM (*n *= 6). *P <0.05 and **P <0.01 as compared to mice treated with carrageenan alone.

To determine if the anti-inflammatory properties of muraglitazar are also translated to *in vivo *situations, we examined the effect of muraglitazar on carrageenan-induced inflammatory paw oedema in mice. Muraglitazar prevented the development of oedema in a dose-dependent manner (Figure 7). With the highest dose of muraglitazar (50 mg/kg) used, carrageenan-induced oedema was reduced by 54% at the six-hour time point. Dexamethasone 2 mg/kg and iNOS inhibitor L-NIL 50 mg/kg were used as control compounds and they reduced the oedema by 69% and 48%, respectively.

**Figure 8:**
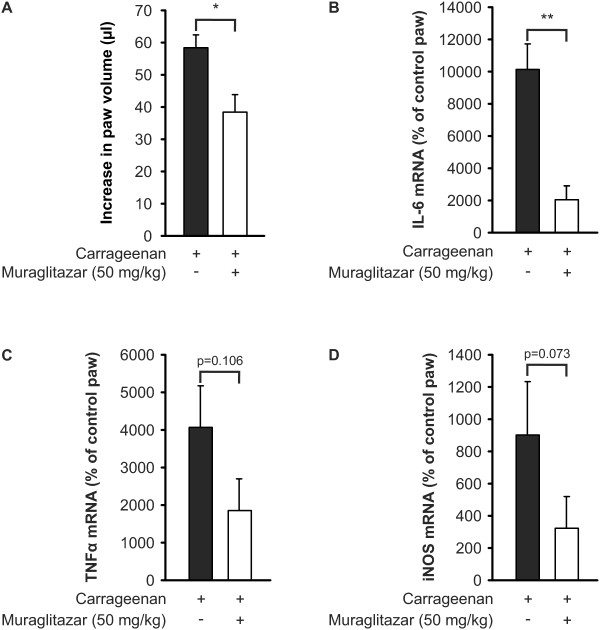
**Effect of muraglitazar on carrageenan-induced production of inflammatory mediators in the mouse**. Muraglitazar was administrated to animals intraperitoneally two hours prior to carrageenan injection (1.5%). (**a**) Paw oedema was measured before and six hours after carrageenan injection by plethysmometer. Oedema is expressed as difference between carrageenan-treated paw and control paw. (**b-d**) Six hours after carrageenan injection, the animals were sacrificed and total RNA was extracted from subcutaneous connective tissue of carrageenan-injected and control paw. IL-6, TNFα and inducible nitric oxide synthase (iNOS) mRNA were determined by RT-PCR. The results were normalized against glyceraldehyde-3-phosphate dehydrogenase (GAPDH) mRNA and mRNA levels in carrageenan-treated paw were compared to those in control paw. Values are mean + SEM (*n *= 5 to 7). *P <0.05 and **P <0.01 as compared to mice treated with carrageenan alone.

In the following series of experiments, we analysed the effect of muraglitazar on inflammatory gene expression in carrageenan-induced inflammation. In line with the previous series, muraglitazar attenuated carrageenan-induced paw oedema (Figure 8a). Muraglitazar also decreased the levels of IL-6, TNFα and iNOS mRNA by 80%, 54% and 64%, respectively (Figure 8b-d).

## Discussion

The present findings show that muraglitazar has anti-inflammatory properties both in *in vitro *and *in vivo *models. The results suggest that the anti-inflammatory effects of muraglitazar reflect the agonistic action through both PPARα and PPARγ. To our knowledge, this is the first report describing the effects of dual PPARγ/α agonists on inflammatory responses.

In support of the present findings, previous reports also describe the inhibitory effects of PPARγ or PPARα agonists on inflammatory mediators in cell culture experiments [22]. PPARγ and PPARα ligands have been reported to decrease plasma levels of proinflammatory cytokines in diabetic and hyperlipidemic patients [23]. In our experiments, PPARα and PPARγ agonists had somewhat different effects on IL-6, TNFα and iNOS expression. All the agonists reduced IL-6 production in a similar potency. However, fenofibrate was not able to decrease TNFα production or iNOS mRNA levels, contrary to the effects of GW1929 or muraglitazar. On the other hand, unlike GW1929, muraglitazar and fenofibrate were able to decrease iNOS protein levels still at post-transcriptional time points. This suggests that activation of PPARα and PPARγ regulate different pathways in the LPS-activated inflammatory cascades. The effects of PPAR agonists on iNOS expression are supported by our previous findings showing that PPARα agonists WY14643 and GW7647 regulate iNOS expression by enhancing its degradation [19]. The present results imply that the effect of muraglitazar on TNFα production is mediated by the PPARγ component of action and on IL-6 production and iNOS expression by both PPARα and PPARγ components of action. Especially, as for the inhibition of iNOS expression, muraglitazar seems to be a more potent inhibitor than fenofibrate or GW1929 alone. This is explained by the findings that PPARγ (but not PPARα) activation inhibits iNOS transcription and PPARα (but not PPARγ) activation has an effect at post-transcriptional level, while muraglitazar (as expected) has both of those effects. Thus the results suggest that combining the effects of PPARα and PPARγ by using a dual PPARγ/α agonist results in improved anti-inflammatory action as compared to PPARα or PPARγ agonists alone.

In the present study, muraglitazar reduced iNOS mRNA expression and promoter activity but did not affect the activity of NF-κB, which is an important transcription factor for iNOS and IL-6 genes. According to our preliminary experiments, muraglitazar did not affect the activation of STAT1 or IRF1 either. Although the activity of NF-κB was not altered, it is possible that muraglitazar regulates the transcription machinery in a more complicated manner. Still, further studies are needed to uncover the detailed target of muraglitazar in the transcriptional activation of iNOS.

There is evidence suggesting that PPARγ and PPARα agonists attenuate experimentally induced arthritis in murine models. Tomita *et al*. [24] and Sumariwalla *et al*. [25] showed that PPARγ agonists THR0921 and CLX-090717, respectively, reduced clinical signs of synovitis in collagen-induced arthritis in the mouse. THR0921 also decreased the circulating levels of IgG antibody to the collagen used in immunization [24]. Koufany *et al*. [26] reported that thiazolidinediones rosiglitazone and pioglitazone reduced synovial expression of TNFα, IL-1β and basic fibroblast growth factor and clinical signs of synovitis in Freund's adjuvant-induced arthritis in rats. However, less is known about the possible effects of PPARα agonists in inflammation models. Okamoto *et al*. [12] showed that fenofibrate decreased signs of arthritis and, to a lesser extent, paw oedema in Freund's adjuvant-induced arthritis in rats.

In the present study, we extend the previous data by showing that muraglitazar significantly decreased carrageenan-induced paw inflammation in mice. Carrageenan-induced paw oedema is a widely used model in inflammation research. It represents features of acute inflammation and innate immune responses and is useful for primary screening of therapeutic efficacy of novel anti-inflammatory agents [27]. Carrageenan-induced inflammation has been described to occur in two phases. The first and short-lasting phase starts almost immediately after the injection of carrageenan. The inflammation during this phase is local and the most abundant mediators are histamine, serotonin and kinins. The second phase starts in one to two hours after carrageenan injection and has been described to involve the activation of inflammatory cells, including macrophages. The expression of iNOS and COX-2, and the synthesis of prostaglandins, oxygen-derived free radicals, TNFα, IL-1β and IL-6 are increased in the inflamed tissue [28-30]. In our experiments, muraglitazar did not reduce paw oedema at early time points, that is, during the first phase of inflammation, while the anti-inflammatory effects at later time points were clear. The results of the effects of muraglitazar on paw oedema and inflammatory gene expression in mice are in line with our results *in vitro*, where muraglitazar reduced the production of NO, IL-6 and TNFα and the expression of iNOS. These results show that muraglitazar has anti-inflammatory potency also *in vivo*.

During the last years the safety aspects of PPAR agonists have been discussed. Rosiglitazone was withdrawn from the market in Europe in September 2010, since it had been found to increase the risk of myocardial infarction in diabetic patients [31]. Fibrates, on the other hand, were suggested to increase the risk of myopathy, hepatotoxicity, cholecystitis and deep venous thrombosis [32]. However, in detailed studies, the increased risk of myocardial infarction has specifically been linked to rosiglitazone [33], while the other thiazolidinedione on the market, pioglitazone, seems to be a rather safe drug [34,35]. In addition, fibrates did not show increased risk of serious drug-related adverse events in a recently published meta-analysis when they were used as a monotherapy for the treatment of dyslipidemia [36]. Thus, it seems so far that the side effects of PPAR agonists are compound-dependent and not associated with the mechanism of action, and the advantages of these drugs in diabetes and dyslipidemia are markedly greater than the possible disadvantages. Muraglitazar has been reported to have similar cardiovascular side effects as rosiglitazone, at least when combined with sulfonylureas or metformin [37]. Because of the adverse effects, muraglitazar's developer Bristol-Myers Squibb decided to discontinue further development of the drug in May 2006. Nevertheless, although the safety data published need to be considered, it is worth noticing that only little knowledge of using muraglitazar in nondiabetic patients is available. Thus, further studies are needed to establish the safety of muraglitazar and other dual PPARγ/α agonists in other possible indications than diabetes.

The connection between metabolism and inflammation is interesting and there is lots of research activity going on in that area at this time. As many studies have already suggested, PPARs might be an important link between these two complex systems, and as shown in the present study, PPAR agonists have anti-inflammatory properties in classical inflammatory models. It has been shown that a good treatment of metabolic diseases reduces the low-grade inflammation associated with obesity [38]. But could the modification of metabolic pathways reduce the inflammatory responses associated to classical inflammatory diseases? This is a question we will be interested in answering in the future.

## Conclusions

The present study shows that muraglitazar has several anti-inflammatory effects in activated macrophages and in carrageenan-induced inflammation in the mouse, reflecting its activity on both PPARα and PPARγ. The results strengthen the previous evidence of the connection of metabolism and inflammation. Understanding this connection in more detail might open a new avenue in the treatment of chronic inflammatory diseases in the future.

## Abbrevations

CMC: carboxymethyl cellulose; DMSO: dimethyl sulfoxide; EI: electron ionization; ESI: electrospray ion source; FTICR: Fourier transform ion cyclotron resonance; GAPDH: glyceraldehyde-3-phosphate dehydrogenase; IL-6: interleukin 6; iNOS: inducible nitric oxide synthase; L-NIL: N^6^-(1-iminoethyl)-L-lysine; LPS: lipopolysaccharide; NF-κB: nuclear factor kappa B; NO: nitric oxide; PDTC: pyrrolidine dithiocarbamate; PPAR: peroxisome proliferator-activated receptor; SEM: standard error of mean; TBS: tri-buffered saline; TNFα: tumour necrosis factor α; TZDs: thiazolidinediones

## Competing interests

The authors declare that they have no competing interests.

## Author's contributions

EP carried out most of the experiments, participated in the design of the study and interpretation of the results, and drafted the manuscript. TL and ML participated in the experimental analyses and design of the study. MFY and MZA carried out the first series (Figure 7) of the *in vivo *assays. AK and PHA synthesized muraglitazar. EM conceived and coordinated the study, and supervised its design and conduction and writing of the manuscript. All authors read and approved the final manuscript.

## References

[B1] Hamilton JA Tak PP The dynamics of macrophage lineage populations in inflammatory and autoimmune diseases Arthritis Rheum 2009 15 1210 1221 10.1002/art.24505 19404968

[B2] Gierut A Perlman H Pope RM Innate immunity and rheumatoid arthritis Rheum Dis Clin North Am 2010 15 271 296 10.1016/j.rdc.2010.03.004 20510234 PMC2937347

[B3] Zeyda M Stulnig TM Adipose tissue macrophages Immunol Lett 2007 15 61 67 10.1016/j.imlet.2007.07.003 17719095

[B4] Schenk S Saberi M Olefsky JM Insulin sensitivity: modulation by nutrients and inflammation J Clin Invest 2008 15 2992 3002 10.1172/JCI34260 18769626 PMC2522344

[B5] Mathieu P Lemieux I Despres JP Obesity, inflammation, and cardiovascular risk Clin Pharmacol Ther 2010 15 407 416 10.1038/clpt.2009.311 20200516

[B6] Lago F Dieguez C Gomez-Reino J Gualillo O Adipokines as emerging mediators of immune response and inflammation Nat Clin Pract Rheumatol 2007 15 716 724 10.1038/ncprheum0674 18037931

[B7] Shapiro H Lutaty A Ariel A Macrophages, meta-inflammation, and immuno-metabolism ScientificWorldJournal 2011 15 2509 2529 22235182 10.1100/2011/397971 PMC3253544

[B8] Gordon S Alternative activation of macrophages Nat Rev Immunol 2003 15 23 35 10.1038/nri978 12511873

[B9] Fruchart JC Peroxisome proliferator-activated receptor-alpha (PPARα): At the crossroads of obesity, diabetes and cardiovascular disease Atherosclerosis 2009 15 1 8 10.1016/j.atherosclerosis.2009.03.008 19386311

[B10] Fuentes L Roszer T Ricote M Inflammatory mediators and insulin resistance in obesity: role of nuclear receptor signaling in macrophages Mediators Inflamm 2010 15 219583 20508742 10.1155/2010/219583 PMC2874923

[B11] Giaginis C Giagini A Theocharis S Peroxisome proliferator-activated receptor-γ (PPAR-γ) ligands as potential therapeutic agents to treat arthritis Pharmacol Res 2009 15 160 169 10.1016/j.phrs.2009.02.005 19646655

[B12] Okamoto H Iwamoto T Kotake S Momohara S Yamanaka H Kamatani N Inhibition of NF-κB signaling by fenofibrate, a peroxisome proliferator-activated receptor-α ligand, presents a therapeutic strategy for rheumatoid arthritis Clin Exp Rheumatol 2005 15 323 330 15971419

[B13] Devasthale PV Chen S Jeon Y Qu F Shao C Wang W Zhang H Cap M Farrelly D Golla R Grover G Harrity T Ma Z Moore L Ren J Seethala R Cheng L Sleph P Sun W Tieman A Wetterau JR Doweyko A Chandrasena G Chang SY Humphreys WG Sasseville VG Biller SA Ryono DE Selan F Hariharan N Cheng PT Design and synthesis of N-[(4-Methoxyphenoxy)carbonyl]-N-[[4-[2-(5-methyl-2-phenyl-4-oxazolyl)ethoxy]phenyl]methyl]glycine [Muraglitazar/BMS-298585], a novel peroxisome proliferator-activated receptor α/γ dual agonist with efficacious glucose and lipid-lowering activities J Med Chem 2005 15 2248 2250 10.1021/jm0496436 15771468

[B14] Buse JB Rubin CJ Frederich R Viraswami-Appanna K Lin KC Montoro R Shockey G Davidson JA Muraglitazar, a dual (α/γ) PPAR activator: a randomized double-blind, placebo-controlled, 24-week monotherapy trial in adult patients with type 2 diabetes Clin Ther 2005 15 1181 1195 10.1016/j.clinthera.2005.08.005 16199244

[B15] Kendall DM Rubin CJ Mohideen P Ledeine JM Belder R Gross J Norwood P O'Mahony M Sall K Sloan G Roberts A Fiedorek FT DeFronzo RA Improvement of glycemic control, triglycerides, and HDL cholesterol levels with muraglitazar, a dual (α/γ) peroxisome proliferator-activated receptor activator, in patients with type 2 diabetes inadequately controlled with metformin monotherapy Diabetes Care 2006 15 1016 1023 10.2337/dc05-1146 16644631

[B16] Kleinert H Euchenhofer C Ihrig-Biedert I Förstermann U Glucocorticoids inhibit the induction of nitric oxide synthase II by down-regulating cytokine-induced activity of transcription factor nuclear factor-κB Mol Pharmacol 1996 15 15 21 8569701

[B17] Green LC Wagner DA Glogowski J Skipper PL Wishnok JS Tannenbaum SR Analysis of nitrate, nitrite, and [15N]nitrate in biological fluids Anal Biochem 1982 15 131 138 10.1016/0003-2697(82)90118-X 7181105

[B18] Bradford MM A rapid and sensitive method for the quantitation of microgram quantities of protein utilizing the principle of protein-dye binding Anal Biochem 1976 15 248 254 942051 10.1016/0003-2697(76)90527-3

[B19] Paukkeri EL Leppänen T Sareila O Vuolteenaho K Kankaanranta H Moilanen E PPARα agonists inhibit nitric oxide production by enhancing iNOS degradation in LPS-treated macrophages Br J Pharmacol 2007 15 1081 1091 17891158 10.1038/sj.bjp.0707477 PMC2095111

[B20] Kleinert H Schwarz PM Föstermann U Regulation of the expression of inducible nitric oxide synthase Biol Chem 2003 15 1343 1364 14669979 10.1515/BC.2003.152

[B21] Brown KD Claudio E Siebenlist U The roles of the classical and alternative nuclear factor-κB pathways: potential implications for autoimmunity and rheumatoid arthritis Arthritis Res Ther 2008 15 212 10.1186/ar2457 18771589 PMC2575629

[B22] Moraes LA Piqueras L Bishop-Bailey D Peroxisome proliferator-activated receptors and inflammation Pharmacol Ther 2006 15 371 385 10.1016/j.pharmthera.2005.08.007 16168490

[B23] Libby P Plutzky J Inflammation in diabetes mellitus: role of peroxisome proliferator-activated receptor-α and peroxisome proliferator-activated receptor-γ agonists Am J Cardiol 2007 15 27B 40B 17307056 10.1016/j.amjcard.2006.11.004

[B24] Tomita T Kakiuchi Y Tsao PS THR0921, a novel peroxisome proliferator-activated receptor gamma agonist, reduces the severity of collagen-induced arthritis Arthritis Res Ther 2006 15 R7 10.1186/ar1856 16356194 PMC1526548

[B25] Sumariwalla PF Palmer CD Pickford LB Feldmann M Foxwell BM Brennan FM Suppression of tumour necrosis factor production from mononuclear cells by a novel synthetic compound, CLX-090717 Rheumatology 2009 15 32 38 19015145 10.1093/rheumatology/ken398

[B26] Koufany M Moulin D Bianchi A Muresan M Sebillaud S Netter P Weryha G Jouzeau JY Anti-inflammatory effect of antidiabetic thiazolidinediones prevents bone resorption rather than cartilage changes in experimental polyarthritis Arthritis Res Ther 2008 15 R6 10.1186/ar2354 18199331 PMC2374462

[B27] Vogel HG Paw edema Drug discovery and Evaluation: Pharmacological Assays 2002 2 New York: Springer 759 762

[B28] Salvemini D Wang ZQ Wyatt PS Bourdon DM Marino MH Manning PT Currie MG Nitric oxide: a key mediator in the early and late phase of carrageenan-induced rat paw inflammation Br J Pharmacol 1996 15 829 838 10.1111/j.1476-5381.1996.tb15475.x 8799551 PMC1909531

[B29] Loram LC Fuller A Fick LG Cartmell T Poole S Mitchell D Cytokine profiles during carrageenan-induced inflammatory hyperalgesia in rat muscle and hind paw J Pain 2007 15 127 136 10.1016/j.jpain.2006.06.010 16949880

[B30] Morris CJ Carrageenan-induced paw edema in the rat and mouse Methods Mol Biol 2003 15 115 121 12769480 10.1385/1-59259-374-7:115

[B31] Nissen SE Wolski K Effect of rosiglitazone on the risk of myocardial infarction and death from cardiovascular causes N Engl J Med 2007 15 2457 2471 10.1056/NEJMoa072761 17517853

[B32] Davidson MH Armani A McKenney JM Jacobson TA Safety considerations with fibrate therapy Am J Cardiol 2007 15 3C 18C 17368275 10.1016/j.amjcard.2006.11.016

[B33] Home PD Pocock SJ Beck-Nielsen H Curtis PS Gomis R Hanefeld M Jones NP Komajda M McMurray JJ RECORD Study Team Rosiglitazone evaluated for cardiovascular outcomes in oral agent combination therapy for type 2 diabetes (RECORD): a multicentre, randomised, open-label trial Lancet 2009 15 2125 2135 10.1016/S0140-6736(09)60953-3 19501900

[B34] Lincoff AM Wolski K Nicholls SJ Nissen SE Pioglitazone and risk of cardiovascular events in patients with type 2 diabetes mellitus: a meta-analysis of randomized trials JAMA 2007 15 1180 1188 10.1001/jama.298.10.1180 17848652

[B35] Dormandy J Bhattacharya M van Troostenburg de Bruyn AR PROactive investigators Safety and tolerability of pioglitazone in high-risk patients with type 2 diabetes Drug Saf 2009 15 187 202 10.2165/00002018-200932030-00002 19338377

[B36] Jun M Foote C Lv J Neal B Patel A Nicholls SJ Grobbee DE Cass A Chalmers J Perkovic V Effects of fibrates on cardiovascular outcomes: a systematic review and meta-analysis Lancet 2010 15 1875 1884 10.1016/S0140-6736(10)60656-3 20462635

[B37] Nissen SE Wolski K Topol EJ Effect of muraglitazar on death and major adverse cardiovascular events in patients with type 2 diabetes mellitus JAMA 2005 15 2581 2586 10.1001/jama.294.20.joc50147 16239637

[B38] Kontunen P Vuolteenaho K Nieminen R Lehtimaki L Kautiainen H Kesaniemi Y Ukkola O Kauppi M Hakala M Moilanen E Resistin is linked to inflammation, and leptin to metabolic syndrome, in women with inflammatory arthritis Scand J Rheumatol 2011 15 256 262 10.3109/03009742.2010.548827 21453187

